# Genome-centric evaluation of *Burkholderia* sp. strain SRS-W-2-2016 resistant to high concentrations of uranium and nickel isolated from the Savannah River Site (SRS), USA

**DOI:** 10.1016/j.gdata.2017.02.011

**Published:** 2017-02-27

**Authors:** Ashish Pathak, Ashvini Chauhan, Paul Stothard, Stefan Green, Mark Maienschein-Cline, Rajneesh Jaswal, John Seaman

**Affiliations:** a Environmental Biotechnology and Genomics Laboratory, School of the Environment, 1515 S. Martin Luther King Jr. Blvd., Suite 305B, FSH Science Research Center, Florida A&M University, Tallahassee, FL 32307, USA; b Department of Agricultural, Food and Nutritional Science, University of Alberta, Edmonton, AB T6G2P5, Canada; c DNA Services Facility, University of Illinois at Chicago, Chicago, IL 60612, USA; d Savannah River Ecology Laboratory, University of Georgia, Aiken, SC 29802, USA

**Keywords:** Uranium, nickel, Biomineralization, Metal resistance genes, Whole genome sequencing (WGS), *Burkholderia*

## Abstract

Savannah River Site (SRS), an approximately 800-km^2^ former nuclear weapons production facility located near Aiken, SC remains co-contaminated by heavy metals and radionuclides. To gain a better understanding on microbially-mediated bioremediation mechanisms, several bacterial strains resistant to high concentrations of Uranium (U) and Nickel (Ni) were isolated from the Steeds Pond soils located within the SRS site. One of the isolated strains, designated as strain SRS-W-2-2016, grew robustly on both U and Ni. To fully understand the arsenal of metabolic functions possessed by this strain, a draft whole genome sequence (WGS) was obtained, assembled, annotated and analyzed. Genome-centric evaluation revealed the isolate to belong to the *Burkholderia* genus with close affiliation to *B*. *xenovorans* LB400, an aggressive polychlorinated biphenyl-degrader. At a coverage of 90 ×, the genome of strain SRS-W-2-2016 consisted of 8,035,584 bases with a total number of 7071 putative genes assembling into 191 contigs with an N50 contig length of 134,675 bases. Several gene homologues coding for resistance to heavy metals/radionuclides were identified in strain SRS-W-2-2016, such as a suite of outer membrane efflux pump proteins similar to nickel/cobalt transporter regulators, peptide/nickel transport substrate and ATP-binding proteins, permease proteins, and a high-affinity nickel-transport protein. Also noteworthy were two separate gene fragments in strain SRS-W-2-2016 homologous to the *spoT* gene; recently correlated with bacterial tolerance to U. Additionally, a plethora of oxygenase genes were also identified in the isolate, potentially involved in the breakdown of organic compounds facilitating the strain's successful colonization and survival in the SRS co-contaminated soils. The WGS project of *Burkholderia* sp. strain SRS-W-2-2016 is available at DDBJ/ENA/GenBank under the accession #MSDV00000000.

**Table:** 

Specifications	
Organism	*Burkholderia* sp.
Strain	SRS-W-2-2016
Sequencer or array type	Illumina HiSeq2000
Data format	Processed
Experimental factors	Strain isolated on high concentrations of both Uranium and Nickel
Experimental features	Whole genome sequence analysis, assembly, annotation and comparison
Consent	NA
Sample source location	Savannah River Site, Aiken, SC

## 1 Direct link to deposited data

The Whole Genome Shotgun project of *Burkholderia* sp. strain SRS-W-2-2016 reported in this study has been deposited at DDBJ/ENA/GenBank under the accession MSDV00000000 (https://www.ncbi.nlm.nih.gov/nuccore/MSDV00000000); BioProject: PRJNA356728 (https://www.ncbi.nlm.nih.gov/bioproject/PRJNA356728); BioSample: SAMN06141630 (https://www.ncbi.nlm.nih.gov/biosample/SAMN06141630). The version described in this paper is version MSDV00000000.1.

## 2 Text

### 2.1 Background

Historically, the Savannah River Site (SRS, Aiken, SC) served as the Department of Energy (DOE) nuclear material production facility where metal-clad uranium (U) targets were fabricated for plutonium production [1]. Therefore, U and Ni are the two predominant environmental contaminants within the SRS polluted locations. The practice that was used at SRS from 1954 to 1982 was to release the wastewater from metal plating and fabrication processes directly into Tims Branch, a second-order stream, due to which large quantities of both U (depleted and natural) and Ni are deposited within the riparian sediments of Steeds Pond [2], a pre-SRS farm pond that acted as a natural settling basin [3], [4]. Consequently, sediment concentrations of U and Ni at the Steeds Pond site may exceed 1000 mg/kg [3] and presents a unique opportunity to study genome-enabled mechanisms of environmental microorganisms to resist and bioremediate co-contaminant metals and organics.

### 2.2 Isolation of U and Ni resistant strain SRS-W-2-2016

Several bacterial strains resistant to high concentrations of U and Ni were isolated from soils of the Tims Branch/Steeds Pond area (sites 101, 101S and 104) by serially diluting soil slurries and plating onto Luria Bertani (LB) media supplemented with both U (4.2 mM) and Ni (8.5 mM); these concentrations are similar to those present in the collected SRS soils (data not shown). Plates were incubated aerobically at 30 °C in the dark and colonies that grew within a week were further isolated on LB and LB + U + Ni. One isolate from site 101, tentatively named as strain SRS-W-2-2016, grew robustly on both U and Ni and was chosen for further genome-centric evaluation.

**Fig. 1:**
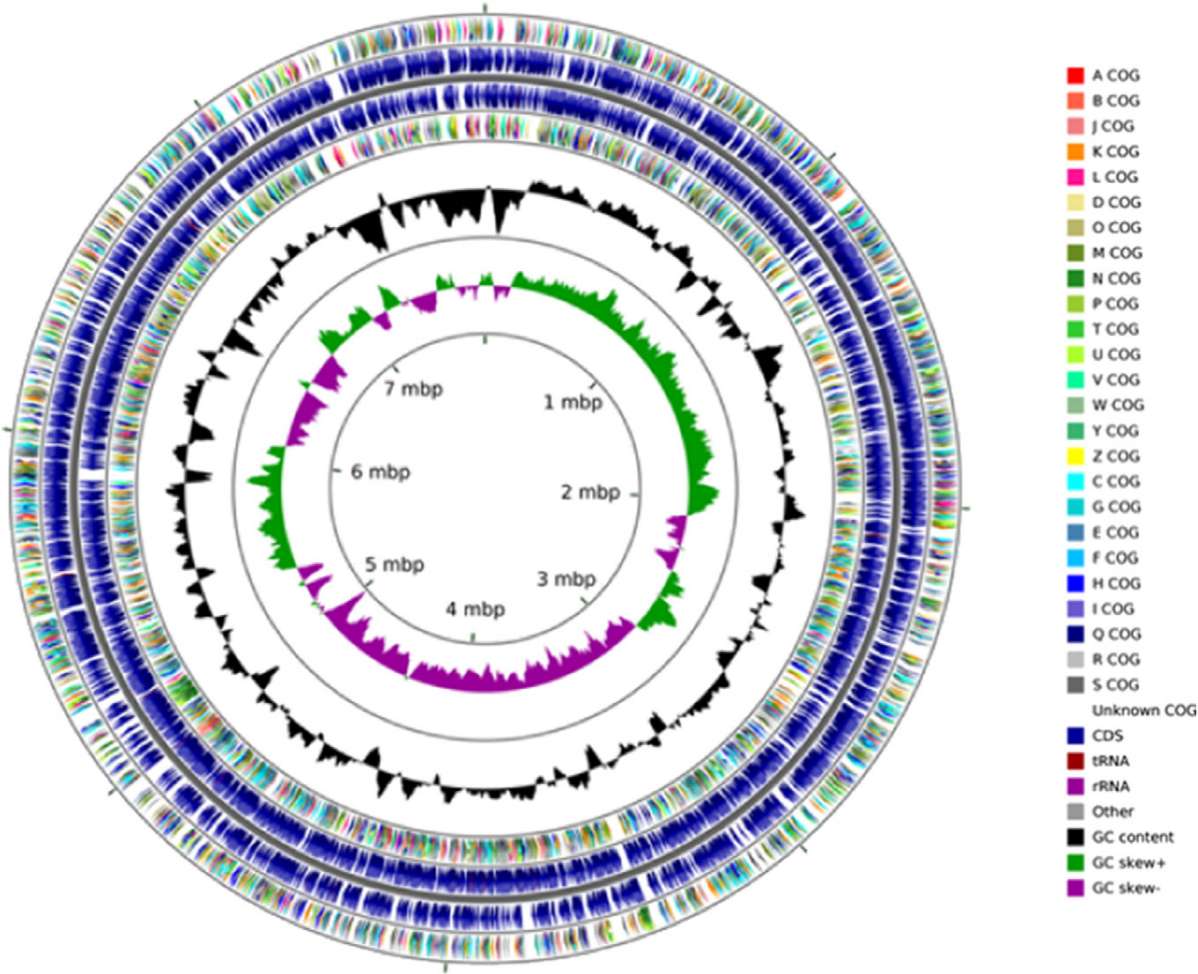
Circular genome map of *Burkholderia* sp. strain SRS-W-2-2016 with the first (outermost) and fourth rings depicting COG categories of protein coding genes on the forward and reverse strands, respectively. The second and third rings show the locations of protein coding, tRNA, and rRNA genes on the forward and reverse strands, respectively. The black plot depicts GC content with the peaks extending towards the outside of the circle representing GC content above the genome average, whereas those extending towards the center mark segments with GC content lower than the genome average. The innermost plot depicts GC skew. Both base composition plots were generated using a sliding window of 50,000 nt.

Briefly, genomic DNA from the strain was extracted and prepared for sequencing on an Illumina HiSeq2000 instrument as described previously [5], [6]. De novo assembly of the raw reads was performed with the SPAdes assembler [7] using default settings. Assembly coverage statistics were computed by mapping raw reads to the assembled genome using bowtie2 [8]; contigs with coverage less than 37.65 were removed from the assembly. The remaining reads were aligned with nucmer [9] against the closest reference sequence from NCBI (determined by a BLAST of the 16S rRNA sequence): accession numbers CP002013.1, CP002014.1, and NC_014119.1 for chromosomes 1, 2, and 3. All contigs aligned to these references, and the optimal contig ordering and orientation to most closely match the reference was determined using mummerplot [9] with–layout specified. Contigs were then reordered and reversed as needed to match the ordering determined by mummerplot. The genome, with a coverage of 90 ×, was then annotated and genes predicted by IMG/er [10], RAST [11] and NCBI's Prokaryotic Genomes Automatic Annotation Pipeline (PGAAP), version 2.0. A circular genomic map of strain SRS-W-2-2016 was generated using CGView [12] using the 191 assembled contigs with N50 contig length of 134,675 bases (Fig. 1), arranged over 96 scaffolds. The genomic size of strain SRS-W-2-2016 was estimated to be approximately 8,035,584 bases with a G + C content of 64.4. Moreover, the strain contained a total of 7071 putative genes, with 96 total RNA genes, 59 tRNA genes and 2 copies of the 16S rRNA genes, respectively.

### 2.3 Genome-centric evaluation of strain SRS-W-2-2016

**Fig. 2:**
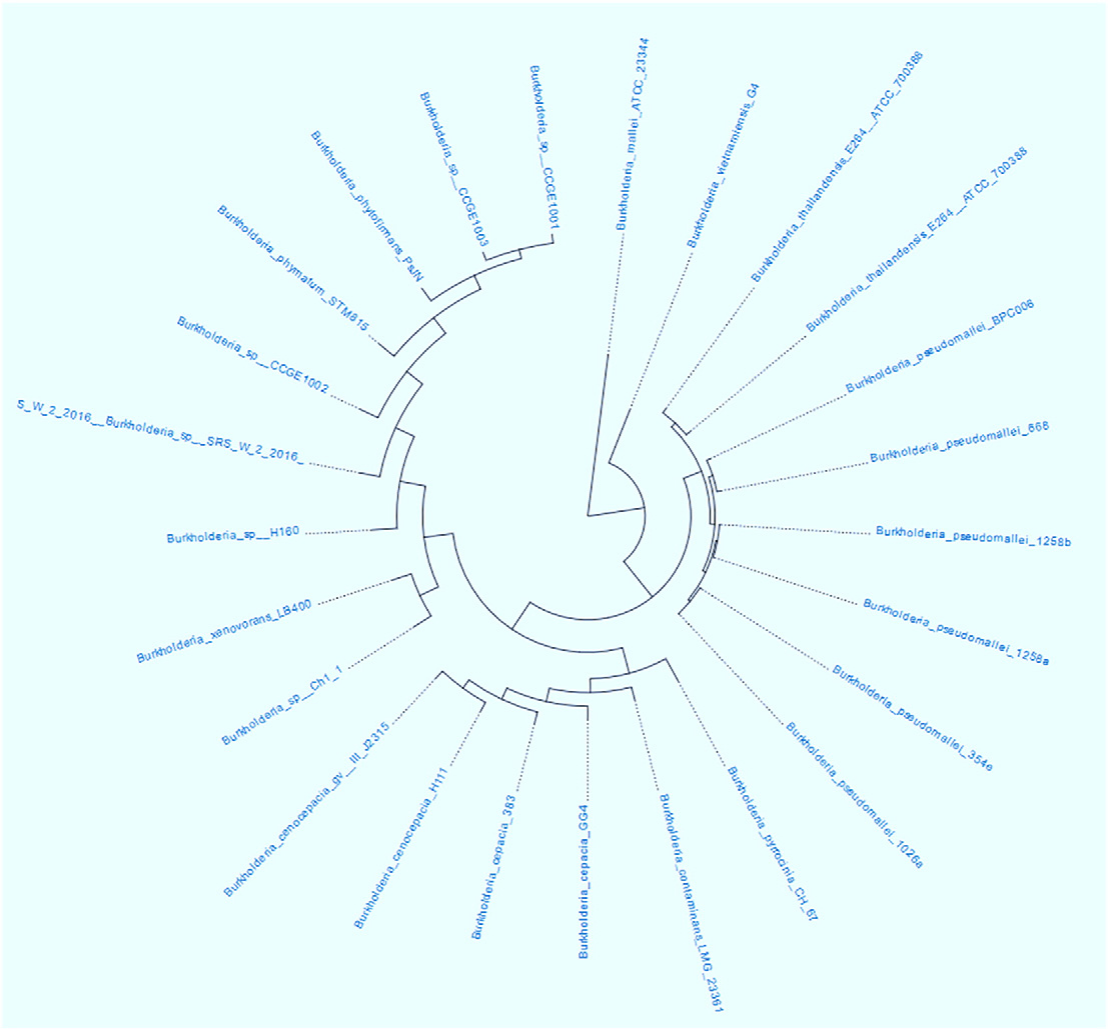
Whole genome sequence based hierarchical clustering analysis phylotree of *Burkholderia* sp. strain SRS-W-2-2016 relative to a cohort of 24 *Burkholderia* spp. The tree was generated based on the presence of Pfam function category amongst *Burkholderia* spp. for which whole genome sequences are available in the IMG/er database.

A further genome-centric evaluation of strain SRS-W-2-2016 revealed that out of 6975 total protein coding genes, 71.06% genes associated with clusters of orthologous groups of proteins (COGs); 78.94% genes annotated to protein coding function predictions; 24.61% genes annotated as protein coding genes with enzyme production and 29.09% genes were connected to KEGG pathways. We then selected the whole genome sequences of 24 other *Burkholderia* strains that are available in IMG/er to run a comparative analysis of strain SRS-W-2-2016 relative to other sequenced *Burkholderia*, which revealed taxonomic affiliation with *Burkholderia* spp. CCGE1002 and H160, respectively (Fig. 2). The *Burkholderia* group of beta-proteobacteria are ubiquitously distributed in diverse ecological habitats ranging from soil to aqueous environments, forming symbiotic associations with plants and animals, serving as saprophytes to endosymbionts and even as biocontrol agents of pathogens [13]. Moreover, a shared trait with many *Burkholderia* species are the presence of a large, multi-replicon genome with a plethora of multiple insertion sequences likely conferring genome plasticity and metabolic versatility to this genus [14]. It is interesting to note that the closest taxonomic relatives of strain SRS-W-2-2016 were isolated from legume nodules [15] and shared approximately 190 CDS related to aromatic compound metabolism along with 150 common CDS for resistance to antibiotics and toxic compounds. Strain SRS-W-2-2016 also affiliated closely with *B*. *xenovorans* LB400, which harbors one of the two largest known bacterial genomes [16]. Strain LB400 is a robust polychlorinated biphenyl-degrader and can also metabolize compounds containing single-carbon (C1) groups, isoflavonoids, diterpenoids, and sulfonates.

**Fig. 3:**
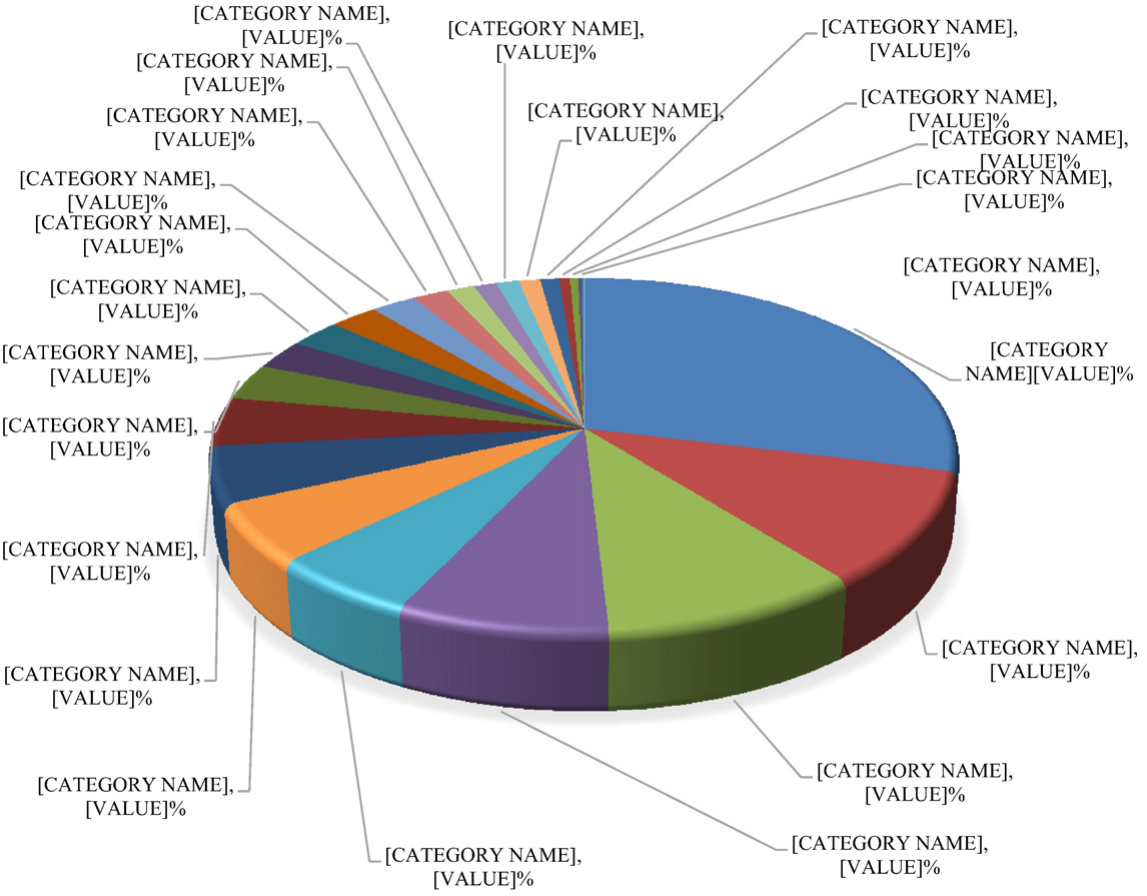
Functional traits connected with KEGG pathways identified from the whole genome sequence of *Burkholderia* sp. strain SRS-W-2-2016. This figure was prepared by analysis of the whole genome using IMG/er and values were plotted using Microsoft Excel.

Furthermore, we performed a KEGG (Kyoto Encyclopedia of Genes and Genomes)-based hierarchical functional gene clustering analysis of strain SRS-W-2-2016 (Fig. 3), which revealed the presence of a total of 2057 KEGG genes with the top 5 categories belonging to amino acid metabolism (419 genes); carbohydrate metabolism (402 genes); membrane transport (327 genes); energy metabolism, (232 genes), and xenobiotics biodegradation and metabolism (218 genes), respectively. Additionally, a total of 5025 protein coding genes associated with COGs in strain SRS-W-2-2016 were found with further classification into 24 categories. The following were most abundant COG subsystems in strain 2385: amino acid transport and metabolism (9.72%); transcription (9.57%); carbohydrate transport and metabolism (8.87%); energy production and conversion (6.84%); cell wall/membrane/envelope biogenesis (6.51%); lipid transport and metabolism (5.74%); signal transduction mechanisms (5.05%); inorganic ion transport and metabolism (4.93%); coenzyme transport and metabolism (4.84%); secondary metabolites biosynthesis, transport and catabolism (3.88%)-many of these features suggest genomic propensity of this strain to engage in high metabolic activity in its native contaminated environment. The strain also contains 11 biosynthetic gene clusters containing 209 genes; a total of 2.96% of its total genome. These analyses suggest *Burkholderia* sp. strain SRS-W-2-2016 to harbor various genome-enabled metabolic and catabolic processes that potentially facilitate its successful colonization and survival within the SRS co-contaminated soils.

### 2.4 Genes potentially conferring metal resistance in strain SRS-W-2-2016

One notable attribute of the *Burkholderia* genus is their vigorous ability to degrade xenobiotic compounds and resistance to heavy metals [15], [17], [18], [19], [20], including U [21] and Ni [22]. Genome-centric assessment of strain SRS-W-2-2016 led to the identification of several gene homologues previously implicated in the resistance against heavy metal/radionuclides, such as the cobalt-zinc-cadmium efflux system, MFS transporters and permeases, outer membrane factor (OMF) lipoprotein, NodT family, multidrug efflux pumps, arsenite efflux membrane protein ArsB, and two-component system, OmpR family and copper resistance phosphate regulon response regulator CusR.

**Fig. 4:**
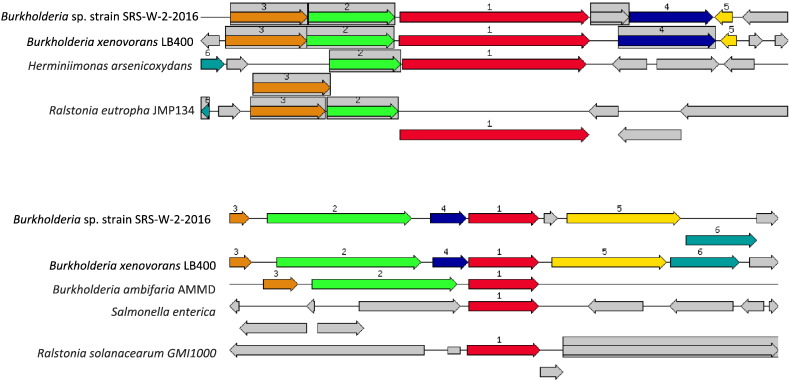
Shown are A, the chromosomal region of the cobalt/zinc/cadmium subsystem gene (1476 bp) in *Burkholderia* sp. strain SRS-W-2-2016 relative to other bacteria and; B, the HoxN/HupN/NixA family nickel/cobalt transporter gene (1029 bp) in *Burkholderia* sp. strain SRS-W-2-2016 relative to other bacteria. The graphic is centered on the focus gene, which is red and numbered 1. Sets of genes with similar sequence are grouped with the same number and color. Genes whose relative position is conserved in at least four other species are functionally coupled and share gray background boxes.

Specifically, the cobalt/zinc/cadmium resistant gene in strain SRS-W-2-2016 was found to be 1476 bp in size (contig24756; fig | 6666666.232708.peg.1176) which is shown relative to four similar organisms (Fig. 4a). We also identified the HoxN/HupN/NixA family high affinity nickel/cobalt transporter-permease gene complex (contig41300; fig | 6666666.232708.peg.2084) which was 1029 bp (Fig. 4b). In addition to these genes, bacterial resistance to metals is also governed by genes for the RND family proteins-these provide for resistance, nodulation, and cell division, respectively-all part of transenvelope protein complexes which detoxify the cellular environment by exporting toxic metal cations from the periplasm to the outside. Several RND-type efflux gene homologues were found interspersed within the genome of strain SRS-W-2-2016 (data not shown). Moreover, the ABC-type transporters, heavy-metal-translocating P-type ATPases, and heavy-metal transport/detoxification proteins were plentifully represented in the genome of strain SRS-W-2-2016, potentially maintaining metal homeostasis.

**Fig. 5:**
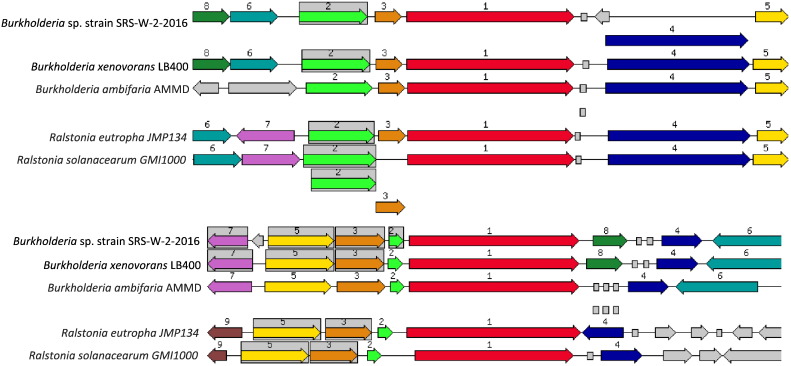
Shown are the chromosomal regions of the ppGpp synthetase/hydrolase (spoT) gene; homologues of TolC in *Escherichia coli* that mediate S-layer export. The SpoT gene was recently implicated in the resistance to U stress in *Caulobacter crescentus*[25]. A, the 2244 bp fragment [GTP pyrophosphokinase (EC 2.7.6.5), (p)ppGpp synthetase I] and; B, the 2361 bp fragment harboring the GTP pyrophosphokinase (EC 2.7.6.5), (p)ppGpp synthetase II/Guanosine-3′,5′-bis(diphosphate) 3′ pyrophosphohydrolase (EC 3.1.7.2) genes, respectively. The graphic is centered on the focus gene, which is red and numbered 1. Sets of genes with similar sequence are grouped with the same number and color. Genes whose relative position is conserved in at least four other species are functionally coupled and share gray background boxes.

One mechanism used by *Burkholderia* to biomineralize metals, especially U, is by the hydrolysis of organophosphate compounds with simultaneous release of extracellular orthophosphate [21], [23], which can convert U(VI) into phosphate-minerals. In fact, microbially-mediated phosphatase activities (e.g., alkaline or acid) can precipitate greater than 90% of soluble U resulting in the formation of a wide array of uranyl phosphate minerals [24]. Therefore, the microbially-mediated formation of uranyl phosphate from U(VI) warrants further research for control of U mobility in U.S. DOE contaminated environments. To identify the genomic basis for phosphatase enzyme activity which may form the basis for U biomineralization by strain SRS-W-2-2016, we manually queried the annotated genome revealing the presence of two acid phosphatases (contig28472, accession # NZ_MSDV01000021.1; contig111382, accession # NZ_MSDV01000039.1) as well as alkaline phosphatase (contig94224; accession # NZ_MSDV01000053.1). Moreover, we also identified the *spoT* gene (contig104637; accession # NZ_MSDV01000038.1) in strain SRS-W-2-2016 (Fig. 5a, b); the *spoT* gene is correlated to a ppGpp hydrolase/synthetase and was recently implicated in the resistance to U tolerance of *Caulobacter crescentus* but only under carbon starvation conditions [25].

In addition to the metal related genes, several genes that potentially confer the ability to degrade organic contaminants were also identified in strain SRS-W-2-2016; especially the oxygenases (data not shown). This is relevant because oxygenases have been previously shown to co-metabolize degradation of trichloroethylene (TCE), one of the widespread organic contaminants identified at the SRS site [26]. It is likely that strain SRS-W-2-2016 is metabolically active in the biodegradation and biomineralization of not only metals but also hydrocarbons in the SRS soils.

### 2.5 Genomic islands (GEIs) in strain SRS-W-2-2016

**Fig. 6:**
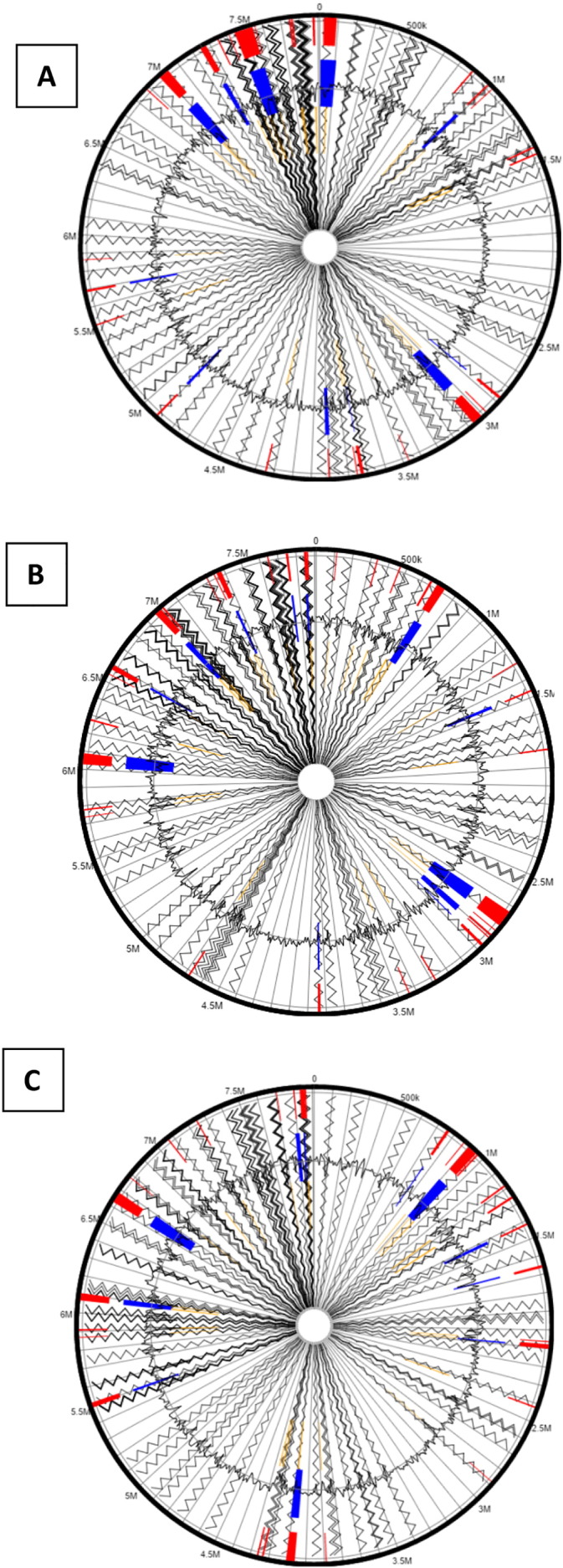
Putative genomic islands (GEIs) predicted within the genome of *Burkholderia* sp. strain SRS-W-2-2016 when aligned against *Burkholderia xenovorans* LB400 chromosome 1 (A), chromosome 2 (B), and chromosome 3 (C) as the reference genomes. The outer black circle represents the scale line in Mbps and the black zig-zag line plot delineates each of the 175 contigs identified from strain SRS-W-2-2016. GEIs obtained from each of the following methods are shown in color: SIGI-HMM (orange), IslandPath-DIMOB (blue), and integrated detection (red), respectively.

Another interesting genomic trait of strain SRS-W-2-2016 are the presence of several genomic islands (GEIs) spread over the three chromosomes that are typical of this genus. Bacterial genomes consist of a core set of genes encoding for essential metabolic functions; these can be augmented with GEIs [27] acquired from the bacterium's native environment by horizontal gene transfer (HGT) mechanisms. The set of foreign genes recruited by the bacteria can provide the host with environmentally adaptive traits and genomic plasticity. GEIs have been more commonly known to render virulence or antibiotic resistance to the host bacteria, but more recently, whole genome sequencing studies have also revealed other adaptive functional traits encoded by GEIs that are classified into the following 4 categories - pathogenicity islands (PAIs), that code for virulence genes; metabolic islands (MIs), genes for biosynthesis of secondary metabolites; resistance islands (RIs), genes that code for resistance-typically against antibiotics; and symbiotic islands (SIs), facilitating symbiotic associations of the host with other micro- and macroorganisms, respectively. We used Island Viewer to identify genomic islands (GEIs) [28] in SRS-W-2-2016, which predicts GEIs integrating two widely used sequence composition based GEI prediction methods-SIGI-HMM and IslandPath-DIMOB along with a comparative GI prediction method-IslandPick. Interestingly, a plethora of genomic islands were identified in SRS-W-2-2016 when compared against the chromosome 1, 2 or 3 of *Burkholderia xenovorans* LB400 as the reference strain (Fig. 6a–c). When GEI-encoded homologues in strain SRS-W-2-2016 were further analyzed by BLAST, several of them closely affiliated with genes previously implicated in the bioremediation of both metals and organic compounds, suggesting that GEIs were horizontally acquired by strain SRS-W-2-2016 from other biodegradative bacteria to facilitate survival in its native environment that is co-contaminated by heavy metals and aromatics.

### 2.6 Conclusions

Genome-centric analysis of heavy metal and radionuclide resistant microbiota isolated from historically contaminated environments, such as the Savannah River Site (SRS), are lacking. SRS is a former nuclear weapons production facility located near Aiken, SC which is co-contaminated with heavy metals, radionuclides and volatile organic compounds (VOCs). Because environmental microorganisms underpin some of the metal transformations, including radionuclide precipitation, sorption, intracellular accumulation and biomineralization, this study is a genome-centric assessment to probe for those genes that facilitate survival in co-contaminated habitats. Towards this end, this study facilitates a deeper understanding of heavy metal and radionuclide resistance of a *Burkholderia* sp. strain SRS-W-2-2016 isolated from a historically co-contaminated soil habitat. Overall, we show that *Burkholderia* sp. strain SRS-W-2-2016, which was isolated on very high concentrations of both Uranium and Nickel, possesses a suite of ecologically relevant genomic traits, to include substrate binding proteins, permeases, transport regulators and efflux pumps-all targeted to continuously shunt metals to the extra-cellular environment or prevent their cellular uptake so the bacterium can persist along with the mixed contaminants. It also appears that horizontal gene transfer is an evolutionary mechanism that equips microbiota to facilitate their colonization and proliferation of radionuclide and hydrocarbon contaminated environments. Studies such as this will provide a better understanding on bioremediation for rehabilitation and stewardship of historically polluted environments. Future studies will involve evaluating gene expression of strain SRS-W-2-2016 when grown in the presence of U, Ni or a combination thereof, which will reveal precise genomic signals that enable microbial resistance and biomineralization mechanisms in co-contaminated habitats such as the SRS.

## 3 Nucleotide sequence accession number

The Whole Genome Shotgun project of *Burkholderia* sp. strain SRS-W-2-2016 reported in this study has been deposited at DDBJ/ENA/GenBank under the accession #MSDV00000000 (https://www.ncbi.nlm.nih.gov/nuccore/MSDV00000000).
